# Functional Disorganization of Small-World Brain Networks in Patients With Ischemic Leukoaraiosis

**DOI:** 10.3389/fnagi.2020.00203

**Published:** 2020-07-03

**Authors:** Yixin Zhu, Tong Lu, Chunming Xie, Qing Wang, Yanjuan Wang, Xuejin Cao, Yuting Su, Zan Wang, Zhijun Zhang

**Affiliations:** ^1^Department of Neurology, Affiliated ZhongDa Hospital, School of Medicine, Southeast University, Nanjing, China; ^2^Department of Radiology, ZhongDa Hospital Affiliated to Southeast University, Nanjing, China

**Keywords:** brain connectome, cognitive impairment, graph-theory, ischemic leukoaraiosis, resting-state fMRI, small world

## Abstract

Cognitive impairment is a key clinical feature of ischemic leukoaraiosis (ILA); however, the underlying neurobiological mechanism is still unclear. ILA has been associated with widespread gray and white matter (WM) damage mainly located in cortical-cortical and cortico-subcortical pathways. A total of 36 patients with ILA (Fazekas rating score ≥2) and 31 healthy controls (HCs) underwent comprehensive neuropsychological assessments (covering four cognitive domains, i.e., information processing speed, episodic memory, executive and visuospatial function) and resting-state functional MRI scans. Graph theory-based analyses were employed to explore the topological organization of the brain connectome in ILA patients, and we further sought to explore the associations of connectome-based metrics and neuropsychological performances. An efficient small-world architecture in the functional brain connectome was observed in the ILA and control groups. Moreover, compared with the HCs, the ILA patients showed increased path length and decreased network efficiency (i.e., global and local efficiency) in their functional brain networks. Further network-based statistic (NBS) analysis revealed a functional-disconnected network in ILA, which is comprised of functional connections linking different brain modules (i.e., default mode, frontoparietal, ventral attention and limbic systems) and connections within single modules (i.e., ventral attention and limbic systems). Intriguingly, the abnormal network metrics correlated with cognitive deficits in ILA patients. Therefore, our findings provide further evidence to support the concept that ILA pathologies could disrupt brain connections, impairing network functioning, and cognition *via* a “disconnection syndrome.”

## Introduction

Ischemic leukoaraiosis (ILA) is characterized by diffuse areas of hypodensity on CT scans or hyperintensities on T2-weighted MRI brain scans. These changes mostly reflect axonal loss and/or demyelination caused by cerebral hypoperfusion or chronic microvascular disease in elderly individuals (Launer et al., 2006). Cognitive impairment, the most common clinical feature associated with ILA, manifests as deficits in thinking ability, memory, executive function, and attention ability (Sultzer et al., 1995; Li et al., 2017). Previous studies have suggested that normal elderly individuals with ILA are at high-risk of further cognitive decline, and the magnitude of the ILA is positively correlated with the severity of cognitive deficits (Ross et al., 2005; Te et al., 2015; Chen et al., 2016). However, the neurobiological pathway underlying cognitive impairment in ILA has not been well illuminated.

Focal gray matter (GM) atrophy in cortical and subcortical regions, such as the dorsolateral prefrontal, parietal and posterior-superior temporal cortices, precuneus, hippocampus, caudate nucleus, and occipital and sensorimotor cortices, has been reported in ILA (Righart et al., 2013; Lambert et al., 2015). Diffusion tensor imaging studies on ILA have demonstrated white matter (WM) changes, especially in the fronto-subcortical circuits (Viana-Baptista et al., 2008; Yuan et al., 2017). Additionally, evidence from functional brain connectome studies on ILA has further shown the involvement of the dysfunction of several networks in ILA, including the default mode network, frontoparietal network, dorsal attention network, salience network and corticostriatal network (Chen et al., 2016, 2019; Liu et al., 2019a,b). These studies have suggested that “brain network dysfunction” may be the best explanatory model for illuminating the neurobiological pathway underlying cognitive impairment in ILA.

The human brain has been widely considered to execute function through integrated networks. Graph theory analysis, widely employed to examine brain network organization, could provide a powerful framework for characterizing the topological architecture of the brain connectome. For example, normal brain networks are characterized in a small-world fashion (i.e., higher local interconnectivity with short path lengths between brain regions), which supports highly segregated and integrated information processing (Salvador et al., 2005; Hagmann et al., 2007; He et al., 2007). Previous brain connectome-based studies have demonstrated the topological disorganization could predict cognitive impairment in Alzheimer’s disease (Dai and He, 2014; Liu et al., 2014) and late-onset depression (Wang et al., 2016). Concerning cerebral small vessel disease (SVD), the most common vascular cause of vascular cognitive impairment, there have been very few studies exploring the topological organization of the whole-brain connectome. Using diffusion-weighted MRI, two previous studies on SVD have reported that topological disorganization of the structural connectome (i.e., reduced global and local efficiency) is associated with cerebral vascular lesions (i.e., lacunes, WM hyperintensities, and microbleeds), and topological disorganization mediates the association between these vascular lesions and cognitive impairment in SVD (Lawrence et al., 2014; Tuladhar et al., 2016). Recently, using functional MRI, Qin et al. (2019) also reported lower global efficiency and higher path length in SVD patients with thalamus lacunes. These brain connectome studies highlighted the importance of connectome-based analyses in understanding the neurobiological mechanism underlying cognitive impairment in SVD. However, SVD demonstrates several types of neuroimaging features on MRI, especially lacunar stroke and leukoaraiosis (i.e., ILA). Importantly, compared with other features, lacunar stroke and leukoaraiosis have consistently been shown to be associated with converted neurological and cognitive symptoms (Román et al., 2002). Therefore, given that the spatial distribution of lacunes and leukoaraiosis plays a more important role in determining the cognitive outcome than other neuroimaging features of SVD, it is meaningful to recruit patients with ILA when conducting studies to investigate the underlying mechanism of cognitive impairment in SVD.

Here, we performed a case-control study to investigate the functional brain connectomic changes present in ILA using resting-state functional MRI (R-fMRI) data and graph theory-based and network-based statistic (NBS) approaches. We first investigated whether ILA could disrupt the topological architecture of the functional brain connectome and sought to determine whether those topological alterations are associated with the disruption of functional network connectivity. Then, we sought to examine whether brain connectomics disruptions could explain cognitive impairment in patients with ILA.

## Materials and Methods

### Participants

Sixty-seven individuals (i.e., age ≥50 years), including 36 ILA and 31 healthy control (HC) participants were recruited in this study. All ILA patients met the following criteria: (1) diffuse or confluent hyperintensity lesions (Fazekas rating score ≥2) in the subcortical or periventricular WM on T2-weighted and fluid-attenuated inversion recovery MR images; (2) large cortical or subcortical infarcts (diameter >15 mm); (3) no other neurological disease (e.g., multiple sclerosis, Alzheimer’s disease, Parkinson’s disease, epilepsy, and head trauma); (4) no severe internal diseases (e.g., heart diseases, renal failure diseases, liver diseases, tumor, and other systemic diseases); (5) no neuropsychological disorders or mental disease; (6) WM lesions unrelated to vascular diseases (e.g., immune, demyelination, metabolism, toxicity, infection, and other factors; and (7) no MRI contraindications. Notably, ILA patients admitted for acute lacunar stroke were recruited at least 3 months after the onset of the last stroke. This study was approved by the Research Ethics Committee of ZhongDa Hospital Affiliated to Southeast University (2019ZDSYLL189-P01), and all participants signed written informed consent.

### Neuropsychological Assessment

General cognitive function was evaluated by the Mini-Mental State Examination (MMSE) test. A neuropsychological battery was further administered to assess all participants’ specific cognitive performance in information processing speed, episodic memory, visuospatial and executive function, which is consisted of the auditory verbal learning test (AVLT) and its 20 min delayed recall (AVLT-DR), the logical memory test (LMT) and its 20 min delayed recall (LMT-DR), the Rey-Osterrieth complex figure test (CFT) and its 20 min delayed recall (CFT-DR), the clock drawing test (CDT), the trail-making test-A (TMT-A), the trail-making test-B (TMT-B), the Stroop color-word test (Stroop), the digital symbol substitution test (DSST), the verbal fluency test (VFT), the semantic similarity test (Similarity) and the digital span test (DST).

### Imaging Acquisition

Brain MRI data were acquired on a 3.0 T Siemens Verio scanner (Siemens, Erlangen, Germany) with a 12-channel head coil at the Affiliated ZhongDa Hospital of Southeast University. T1-weighted images were acquired by a 3D magnetization prepared rapid gradient echo sequence: repetition time (TR) = 1,900 ms; echo time (TE) = 2.48 ms; matrix = 256 × 256; flip angle (FA) = 9°; field of view (FOV) = 250 mm × 250 mm; thickness = 1.0 mm; gap = 0 mm. Resting-state functional images were acquired using a gradient-recalled echo-planar imaging sequence: TR/TE = 2,000/25 ms; matrix = 64 × 64; FA = 90°; FOV = 240 mm × 240 mm; thickness = 4.0 mm; gap = 0 mm.

### Imaging Processing

Imaging preprocessing was carried out using Statistical Parametric Mapping (SPM8)^1^ and the Data Processing Assistant for Resting-State fMRI (DPARSF)^2^. The first 10 functional volumes were discarded for scanner stabilization and participant’ adaption to the scanning procedure. The remaining images were corrected for timing differences and motion effects. Next, the individual structural images (T1-weighted MPRAGE images) were coregistered to the mean functional image after motion correction using a linear transformation. The transformed structural images were then segmented into GM, WM, and cerebrospinal fluid using a unified segmentation algorithm. The motion-corrected functional volumes were spatially normalized to the Montreal Neurological Institute space and resampled to 3-mm isotropic voxels using the normalization parameters estimated during the unified segmentation process. Further preprocessing included linear detrending and temporal bandpass filtering (i.e., 0.01–0.1 Hz), which were applied to reduce the effects of low-frequency drift and high-frequency physiological noise, respectively. Finally, nuisance signals (six head motion parameters, mean global signal, WM signal, and cerebrospinal fluid signal) were extracted and regressed out from the data. Notably, two ILA patients were excluded as a result of excessive motion artifacts (i.e., a displacement ≥3 mm or an angular rotation ≥3° in all directions). Thus, the remaining 34 ILA patients and 31 HC subjects were included in further analyses.

### Network Construction

Brain regions in each participant were first parcellated into 90 regions using the Automated Anatomically Labeling (AAL) atlas (Tzourio-Mazoyer et al., 2002). Pearson correlation coefficients between every pair of regional time series were then calculated; thus generating a 90 × 90 correlation matrix for each participant. Finally, the matrix was thresholded into a binary graph with a fixed sparsity level for further analyses with graph theory-based approaches (Wang et al., 2016). As there is no gold standard for selecting a single threshold, we applied a wide range of sparsity values (i.e., 10%–40%) in steps of 1%.

### Network Analysis

We performed a whole-brain network analysis using the GRETNA toolbox^3^ (Wang et al., 2015). To characterize the functional brain connectome, we calculated global network measures, such as characteristic path length *L_p_*, clustering coefficient *C*_p_, local efficiency *E*_loc_ and global efficiency *E*_glob_. Briefly, the small-worldness of a network is traditionally characterized by *C*_p_ and *L*_p_ (Watts and Strogatz, 1998). To estimate the small-world properties, we divided *C*_p_ and *L*_p_ by the values obtained from 100 random networks that preserved the same number of nodes, edges, and degree distributions as the real networks (Maslov and Sneppen, 2002; Milo et al., 2002). A small-world network was therefore defined as having a relatively high *C*_p_ (i.e., γ = *C*_p_/*C*_p_^rand^ > 1) and an approximately equivalent *L*_p_ (i.e., λ = *L*_p_/*L*_p_^rand^ ≈ 1) concerning a random network; resulting in a small-worldness scalar σ = γ/λ of more than 1. As graph theoretical approaches have developed, alternative metrics based on network efficiency (Latora and Marchiori, 2001) have received growing attention. The local efficiency *E*_loc_ quantifies the extent of local cliquishness of information transfer of a network, and the global efficiency *E*_glob_ quantifies the parallel information propagation ability of a network. High local and global efficiencies indicate more efficient information transmission over a local and global network, respectively. Details about these network measures were described in previous studies (Rubinov and Sporns, 2010). Besides, the area under the curve (AUC) for each network metric, which provides a summarized scalar for topological properties independent of single threshold selection, was also calculated in this study (Yin et al., 2016).

Also, the NBS approach was further applied to identify the network disconnection pattern in ILA. As in previous studies (Zhang et al., 2011; Yin et al., 2016), we identified regional pairs showing significant between-group differences in connectivity and then used the NBS method to localize connected networks showing significant alterations in the ILA patients. Permutation tests were used to determine significant levels of altered connectivity networks in the NBS analysis. Briefly, we first detected significant nonzero connections (*P* < 0.05, uncorrected) within each group by performing multiple one-sample *t*-tests in an element-by-element manner on Fisher’s transformed correlation matrices. Then, the nonzero connections within either the patient or the control group were combined into a connection mask. The NBS approach was conducted within the connection mask, where a primary threshold (*P* = 0.05) was first applied to a *t*-statistic (two-sample one-tailed *t-tests*). This *t*-statistic was applied to each link to define a set of suprathreshold links among which any connected components and their sizes (defined as the number of links included in these components) were identified. To estimate the significance of each component, the null distribution of the connected component size was empirically derived using a nonparametric permutation approach (10,000 permutations). For each permutation, all subjects were reallocated randomly into two groups, and the *t*-statistic was determined independently for each link. Next, the same threshold (i.e., *P* = 0.05) was used to generate suprathreshold links among which the maximal connected component size was recorded. Finally, for a connected component of size M found in the right grouping of controls and patients, the corrected *P*-value was determined by calculating the proportion of the 10,000 permutations for which the maximal connected component was larger than M. Notably, the effects of age, gender and education were removed by a regression analysis before the permutation tests. For a detailed description, see the study by Zalesky et al. (2012). To better interpret the NBS results, we used the standard 7-system template provided by Thomas Yeo et al. (2011). As in previous studies (Baum et al., 2017), to define the *Priori* network modules, we calculated the purity index for the 7-system parcellation and brain regions from the AAL atlas. This measure quantifies the maximum overlap of the AAL labels and functional systems defined by Thomas Yeo et al. (2011). Each AAL label was assigned to a functional system by calculating the nonzero mode of all voxels in each brain region. Subcortical regions were assigned to an eighth, subcortical module. Therefore, the primary modular partition defined by the 90-node networks consisted of eight brain networks: default mode, frontoparietal, ventral attention, dorsal attention, visual, sensorimotor, limbic, and subcortical systems. The primary modular partition defined for the AAL90-node networks is shown in Figure 2A. The process of defining the priori network modules was conducted with the PANDA toolbox^4^ (Cui et al., 2013).

**Figure 1:**
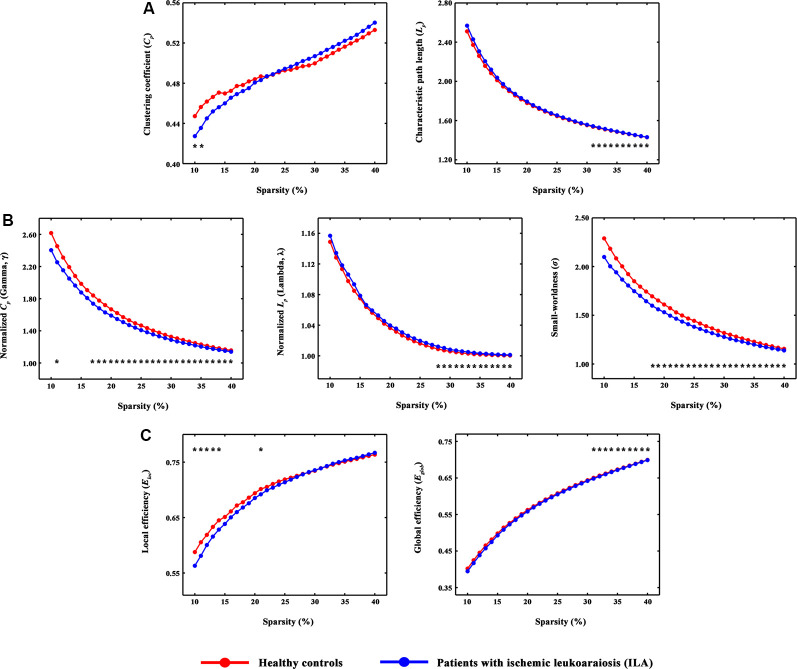
The small-world parameters and network efficiency of whole-brain functional networks. Black asterisks (*) indicate a significant difference between the ILA and HC groups (permutation testing, *P* < 0.05). **(A–C)** The ILA patients showed abnormal global topology in their functional brain networks (i.e., increased absolute and normalized path lengths, and decreased small-worldness and network efficiency), suggesting a less optimal topological organization in ILA. HC, healthy control; ILA, ischemic leukoaraiosis.

**Figure 2:**
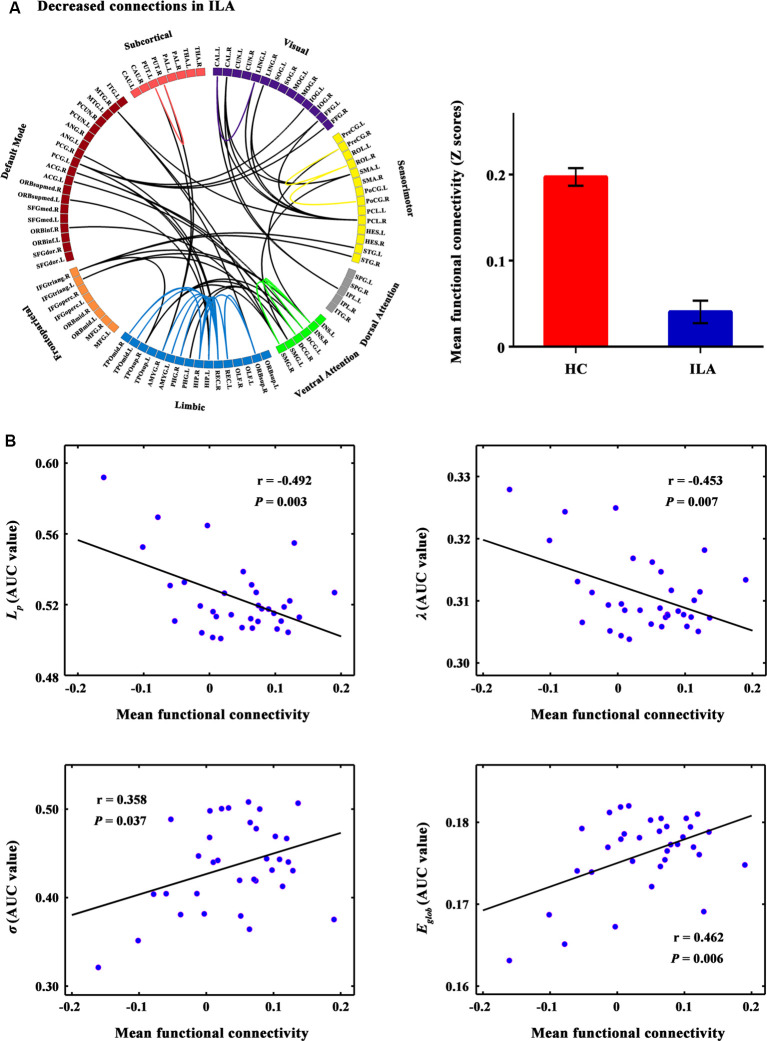
The connected network showing decreased functional connections in ILA patients and their relationships with global network metrics. **(A)** Network-based statistic (NBS) analysis identified a large disconnected network in ILA patients. This network comprises functional connections linking different functional modules (e.g., default mode, frontoparietal, ventral attention, and limbic systems) and connections within single modules (e.g., limbic and ventral attention systems). **(B)** Scatter plots of the mean functional connectivity of this connected network against global network metrics. The mean connectivity value showed significant correlations with four global network metrics (*L_p_*: *r* = −0.492, *P* = 0.003;* λ*: *r* = −0.453, *P* = 0.007; *σ*: *r* = 0.358, *P* = 0.037; *E*_glob_: *r* = 0.462, *P* = 0.006) in the ILA group. ILA, ischemic leukoaraiosis.

### Validation Analysis

We evaluated whether our main results were influenced by head motion. Several recent R-MRI studies have reported influences of head motion on functional connectivity analysis (Power et al., 2012; Van Dijk et al., 2012; Satterthwaite et al., 2013; Yan et al., 2013). In this study, we found that there were no significant between-group differences in the maximum translational and rotational movements and mean frame-wise displacement (Power et al., 2012; all *P*s > 0.05). Nonetheless, we conservatively evaluated the effects of head motion on our results using the “scrubbing” method (Power et al., 2012; Yan et al., 2013). Briefly, we first calculated the frame-wise displacement between the neighboring volumes within each subject and then scrubbed the volumes with a frame-wise displacement above 0.5 mm and their 2 forward and 1 back volume for each subject. Then, between-group differences in topological properties were re-analyzed using the resultant scrubbed R-fMRI data.

### Statistical Analysis

#### Demographic and Neuropsychological Data

The chi-squared and two-independent sample *t*-tests were employed to test the group differences in demographic data. Analysis of covariance (ANCOVAs) were employed to examine the group differences in neuropsychological data, controlling for age, gender, and education as covariates.

#### Network Metrics

Group differences in topological properties were explored by the nonparametric permutation test (Wang et al., 2016). Specifically, group differences in the mean value of each network metric were first calculated. Each subject was then randomly reallocated to one of the two groups and the mean differences between the two randomized groups were recomputed; this process was repeated 10,000 times. The 95th percentile point of the distribution was used as the critical value for a two-tailed test of the null hypothesis. Age, gender, and education were defined as confounding variables.

#### Relationships Between the Network Metrics and Neuropsychological Performances

To examine the clinical relevance of the altered network topologies in ILA patients, we correlated the neuropsychological measures with topological properties at a fixed sparsity (i.e., *S* = 0.4, corresponding to the maximal group difference in *E*_glob_). Partial correlation analyses were conducted, controlling for age, gender, and education as covariates.

#### Exploratory Classification Analysis

To measure the potential of neuroimaging measures for separating the diagnostic groups, combinations of targeted network measures were tested to determine whether they could be used as features that could separate ILA patients from HCs. We took the network measures with significant ILA vs. control group differences as features in the stepwise discrimination analysis. To test the robustness of the results, we also validated the results by using the leave-one-out cross-validation method. This analysis was implemented in SPSS 17.0 (SPSS, Inc., Chicago, IL, USA).

## Results

### Demographic and Neuropsychological Data

Demographics and cognitive performances for all participants are displayed in Table 1. The ILA patients performed worse on the LMT-DR (*P* = 0.022) and DST-backward tests (*P* = 0.039) than the HC subjects. A trend of lower scores on the DST (*P* = 0.052) and Similarity tests (*P* = 0.067) was also observed for the ILA patients. No significant between-group differences were observed in the other neuropsychological performances.

**Table 1:** Demographic and neuropsychological data for all participants.

Item	Healthy controls (*N* = 31)	Patients with ILA (*N* = 34)	*P*-value
**Demographic data**			
Age (years)	60.8 ± 10.7	72.5 ± 7.9	<0.001^a^
Gender (male/female)	16/15	17/17	0.897^b^
Education (years)	11.3 ± 3.3	11.1 ± 3.6	0.824^a^
Smoking (*n*, %)	3 (9.68%)	8 (23.53%)	0.190^c^
Alcohol drinking (*n*, %)	3 (9.68%)	6 (17.65%)	0.480^c^
Hypertension (*n*, %)	6 (19.35%)	24 (70.59)	<0.001^b^
Diabetes (*n*, %)	6 (19.35%)	8 (23.53%)	0.683^b^
**Neuropsychological test data**			
HDRS	2.65 ± 3.66	2.15 ± 3.37	0.607^d^
MMSE	28.42 ± 1.75	26.38 ± 3.68	0.295^d^
Episodic memory			
AVLT-DR	5.90 ± 3.21	3.56 ± 2.79	0.164^d^
LMT-DR	4.35 ± 2.58	2.25 ± 1.88	0.022^d^
CFT-DR	14.42 ± 8.54	7.21 ± 6.37	0.044^d^
Visuospatial function			
CFT	33.48 ± 4.46	31.09 ± 8.99	0.934^d^
CDT	7.97 ± 1.62	7.47 ± 2.00	0.594^d^
Information processing speed			
DSST	41.39 ± 15.42	25.74 ± 13.46	0.052^d^
TMT-A (s)	63.48 ± 25.08	87.47 ± 41.88	0.418^d^
Stroop A (s)	63.48 ± 25.08	87.47 ± 41.88	0.376^d^
Stroop B (s)	40.10 ± 9.96	53.62 ± 16.45	0.184^d^
Executive Function			
VFT-objects	17.94 ± 3.90	16.24 ± 5.46	0.387^d^
VFT-animals	16.19 ± 5.84	15.79 ± 5.17	0.425^d^
DST-backward	4.87 ± 1.36	3.79 ± 1.49	0.039^d^
TMT-B (s)	164.71 ± 92.85	215.91 ± 125.67	0.782^d^
Stroop C (s)	78.55 ± 34.09	109.56 ± 48.41	0.257^d^
Similarity	16.29 ± 4.31	14.06 ± 5.39	0.067^d^

*Data are presented as the mean ± standard deviation. ^a^Independent-sample t-test. ^b\rchi^2^ test. ^c^Fisher’s exact test. ^d^Analysis of covariance (ANCOVA). Abbreviations: ILA, ischemic leukoaraiosis; HDRS, Hamilton Depression Rating Scale; MMSE, Mini-Mental State Examination; AVLT-DR, auditory verbal learning test-20 min delayed recall; LMT-DR, logical memory test-20 min delayed recall; CFT-DR, Rey-Osterrieth complex figure test-20 min delayed recall; CFT, Rey-Osterrieth complex figure test; CDT, clock drawing test; DSST, digital symbol substitution test; TMT-A, trail making test-A; Stroop, Stroop color test; VFT, verbal fluency test; DST, digit span test; TMT-B, trail making test-B; Similarity, semantic similarity test*.

### Network Topological Properties

Over the entire range of sparsity values, the functional brain connectome of the ILA and HC groups showed a higher clustering coefficient *C_p_* (i.e., *γ* > 1) and approximate equivalent path length *L*_p_ (i.e., λ ≈ 1) than those of random networks (Figure 1B). However, further analyses revealed increased path lengths *L*_p_ in the ILA group over a wide range of sparsity values (i.e., 31% ≤ *S* ≤ 40%; Figure 1A). Compared with the HC group, the ILA group (Figure 1B) also showed significantly decreased normalized clustering coefficients *γ* (i.e., 17% ≤ *S* ≤ 40%) and small-worldness (i.e., 18% ≤ *S* ≤ 40%) and increased normalized characteristic path lengths *λ* (i.e., 28% ≤ *S* ≤ 40%). Regarding topological efficiency (Figure 1C), the functional brain connectome of ILA patients showed decreased global efficiency *E*_glob_ (i.e., 31% ≤ *S* ≤ 40%) and local efficiency *E*_loc_ (i.e., 10% ≤ *S* ≤ 14%, *S* = 21%) when compared with those of HC subjects over a range of sparsity. A simple illustration describing the small-world models for ILA and healthy brain networks is shown in Figure 4.

**Figure 3:**
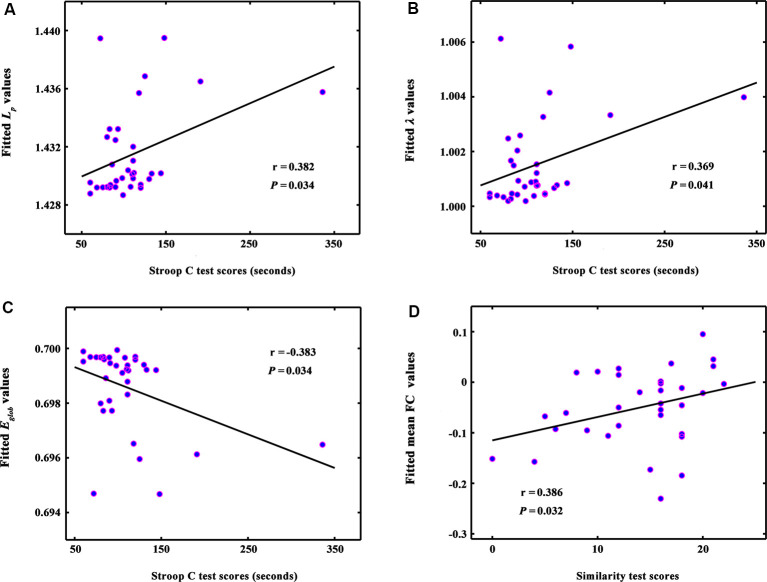
The correlations between global network metrics and cognitive performance in ILA patients. **(A–C)** Within the ILA group, the whole-brain topology (i.e., *L_p_*, *λ*, and *E*_glob_) correlated significantly with the Stroop C scores (*P*s < 0.05). **(D)** The mean functional connectivity strength of the NBS-based connected network exhibited a positive correlation with the Similarity scores (*r* = 0.386, *P* = 0.032). ILA, ischemic leukoaraiosis; NBS, network-based statistic analysis; Stroop C, Stroop color-word test C; Similarity, semantic similarity test.

**Figure 4:**
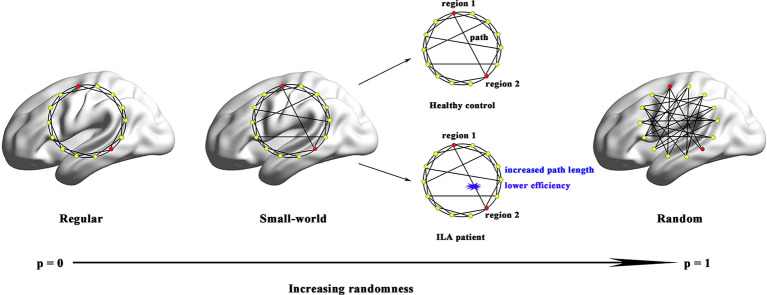
Small-world models for ILA and healthy brain networks. Regular networks have a high local clustering *C_p_* (high local efficiency *E*_loc_) and a long characteristic path length *L*_p_(low global efficiency *E*_glob_). Random networks have a low *C*_p_ (low *E*_loc_) and a small *L*_p_ (high *E*_glob_). The *C*_p_ and *L*_p_ of small-world networks are located between those of regular and random networks. Although both ILA patients and healthy subjects exhibited small-world structure in their functional brain networks, the ILA patients were mainly associated with increased *L*_p_ and lower *E*_glob_.

### Disrupted Functional Network Connectivity in ILA

A disconnected network with 43 nodes and 56 connections was observed in ILA (*P* = 0.031; Figure 2A). This disconnected network was comprised of inter-modular connections, which linked regions in the default mode, frontoparietal, ventral attention, visual and limbic systems, and intra-module connections within the limbic and ventral attention systems. Within the disconnected network, all functional connections showed decreased values in the ILA patients as compared with those in the HC subjects. Intriguingly, the network disconnections showed significant associations with the topological disorganization of the brain connectome in ILA patients (*L*_p_: *r* = −0.492, *P* = 0.003;* λ*: *r* = −0.453, *P* = 0.007; *σ*: *r* = 0.358, *P* = 0.037; *E*_glob_: *r* = 0.462, *P* = 0.006; Figure 2B).

### Relationships Between Network Measures and Neuropsychological Performances

The whole-brain topology (i.e., *L_p_*, *λ*, and *E*_glob_) was significantly correlated with the Stroop C scores (*Ps* < 0.05) in the ILA group (Figures 3A–C). The network disconnections also were positively correlated with the Similarity scores (*r* = 0.386, *P* = 0.032; Figure 3D).

### Validation Results

To determine the extent to which our findings were robust to the motion correction strategy, we repeated the network centrality analyses using the scrubbed R-fMRI data and found that our main results identified in the primary analyses were not affected (Supplementary Figure S1). Note that in this scrubbing analysis, to obtain sufficient time points for stable results, subjects with ≤5 min of data remaining after censoring were excluded from the analysis (one HC and four ILA patient was excluded by this criterion; 60 of 65 participants remained).

### Exploratory Classification Analysis

Considering the relatively small number of subjects included in this study, we used the network measures with significant ILA vs. control group differences as candidate variables. Therefore, six network measures (i.e., *L*_p_, γ, λ, σ, *E*_glob_, and mean NBS-based connectivity) entered into the model. Using this composite model, we could correctly distinguish the ILA patients from the controls in 89.2% of the cases (sensitivity 88.2%, specificity 90.3%). The leave-one-out cross-validation results also showed that 86.2% of the subjects could be correctly classified between the two groups (Table 2). The area under the receiver operating characteristic (ROC) curve (AUC) of the proposed method was 0.94.

**Table 2:** Summary of exploratory classification results of ILA and HC groups.

		Predict group	
	Group	HC	ILA	Accuracy (%)	Ratio (%)	
Original	HC	28	3	89.2	Specificity	90.3
	ILA	4	30		Sensitivity	88.2
Cross-validated^a^	HC	28	3	86.2	Specificity	90.3
	ILA	6	28		Sensitivity	82.4

*^a^Here, we used the leave-one-out cross-validation method. ILA, ischemic leukoaraiosis; HC, healthy control*.

## Discussion

This study investigated the topological organization of the functional brain connectome in ILA patients. Our findings revealed that ILA had aberrant small-world organization and topological efficiency, suggesting a disruption of the normal global integration of brain connectome. Moreover, the ILA-targeted several key disconnections mainly involved the intra-module connections within the ventral attention and limbic systems, and the inter-module connections among different functional systems (i.e., default mode, frontoparietal, ventral attention and limbic systems). Finally, the topological disorganization correlated with cognitive impairment in ILA patients.

The human brain is an interconnected system (or network) that continuously integrates information across brain regions, and has various important topological attributes (Wang et al., 2013). Within the whole-brain network, small-worldness enables highly efficient segregated and integrated information processing (Bullmore and Sporns, 2012). In the present study, small-world topological architecture in functional brain connectomics was observed in HC subjects and ILA patients, implying an optimal organization of the brain to permit highly efficient information transfer and processing. However, quantitative analyses further revealed decreased normalized clustering coefficients and increased absolute and normalized path lengths in the ILA patients relative to those in HC subjects. Network efficiency analyses also showed aberrant small-world topology (i.e., reduced global and local efficiency) in the ILA patients. These results are compatible with previous connectome-based studies on SVD (Lawrence et al., 2014; Tuladhar et al., 2016, 2017; Du et al., 2019).

Given that the small-world architecture reflects an optimal balance between global integration and local specialization (Bullmore and Sporns, 2012), our findings demonstrated a disruption of the normal balance in the whole-brain functional networks of ILA patients. Short path lengths ensure fast information transfer among brain regions that are believed to constitute the basis of cognitive processing (Sporns and Zwi, 2004). Global efficiency is associated with long-range connections and therefore reflects the efficiency of information transfer among remote regions, but local efficiency is predominantly related to short-range connections and therefore indicates the capacity to support specialized processing within densely interconnected brain regions (Wang et al., 2019). Indeed, our findings are supported by many previous neuroimaging studies. For example, microstructural WM disruption has been documented in ILA, such as the frontal WM tracts and corpus callosum (Yamauchi et al., 2000; Jokinen et al., 2007; Viana-Baptista et al., 2008; Wang et al., 2017). Moreover, resting-state functional MRI studies have also shown ILA-related alterations in functional connectivity (Chen et al., 2019; Liu et al., 2019a,b). Therefore, the ILA-related increases in the absolute and normalized path lengths and decreases in the network efficiency might be attributed to disconnections between brain regions.

Intriguingly, we revealed that topological abnormalities (i.e., absolute and normalized path lengths, and global efficiency) correlated with cognitive deficits (i.e., Stroop C scores) in ILA patients. Recently, brain connectome studies on SVD provided evidence that network measures (e.g., network efficiency) could drive the relationship between cerebral vascular lesions and cognitive performance, highlighting the importance of connectome-based analyses in understanding SVD-related cognitive impairment (Du et al., 2019). Therefore, our present study provided further evidence that connectome-based measures could serve as biomarkers monitoring cognitive impairment associated with disease processes.

We further identified a large disconnected network associated with ILA, which is comprised of connections linking different brain modules (i.e., default mode, ventral attention, and limbic systems) and connections within single modules (i.e., ventral attention and limbic systems). Using resting-state functional MRI, a recent study indicated that compared with HCs, SVD patients showed decreased within-network function of the default mode and lower connectivity between the default mode network and other brain networks (i.e., frontoparietal and dorsal attention networks; Liu et al., 2019b). Therefore, our finding is consistent with previous reports of the functional-disconnection of brain networks in SVD. Interestingly, we further found that the network disconnections were correlated to topological disorganization (i.e., absolute and normalized path lengths, small-worldness, and global efficiency). Therefore, we speculate that these network disconnections might lead to a disturbance of the functional brain connectome, subsequently inducing cognitive impairment in ILA patients.

Finally, hypertension and diabetes are very common in elderly individuals and are thought to be vascular risk factors for cognitive impairment (Biessels and Despa, 2018; Iadecola and Gottesman, 2019). In the present study, we found that the proportion of individuals with hypertension was significantly higher among the ILA patients than among the HCs (*P* < 0.001). Given this, we further re-examined the between-group differences in the neuropsychological and network measures, with hypertension and diabetes as additional covariates; unfortunately, our main results could not be reproduced (data not shown). Therefore, we presumed that hypertension and diabetes may be high-risk factors involving the processing of the ILA. Indeed, hypertension and diabetes have been demonstrated to be common risk factors for ILA, and many previous studies have found cognitive impairment and brain structural and functional alterations (i.e., GM atrophy, WM damage, and network dysfunction) in patients with hypertension or diabetes (Reijmer et al., 2013; Biessels and Despa, 2018; Mankovsky et al., 2018; Iadecola and Gottesman, 2019; Tan et al., 2019). Therefore, further studies are needed to be conducted to explore whether brain structural and functional alterations in ILA patients could be mediated by hypertension and/or diabetes.

Several limitations should be addressed. First, this a cross-sectional study, and our sample size was relatively small. Replication of our findings in a large sample will be important for validation. Longitudinal studies are warranted to be performed to investigate the effect of vascular lesions on the conversion from ILA to vascular dementia. Second, given that connectome-based measures are superior to vascular lesions in predicting cognitive impairment in SVD patients (Du et al., 2019), further studies including ILA patients with and without cognitive impairment are important to evaluate whether connectome-based measures could serve as biomarkers for the early identification of ILA patients with cognitive impairment. Third, this was an exploratory study to investigate the cognitive impairment pattern and functional brain connectomic changes in ILA. Our main findings were reported as uncorrected without controlling for multiple comparisons. Therefore, our findings should be interpreted cautiously, and a replication of these findings in a larger cohort will be necessary for validation. Finally, mapping the brain connectome appropriately and precisely is currently a challenging task. We reconstructed the functional brain networks using the Dosenbach-160 atlases (Dosenbach et al., 2010) and found that our main results could be reproduced (Supplementary Figure S2). Nevertheless, previous studies (Wang et al., 2009) have demonstrated the important influences of node choices on the properties of the resulting networks. Therefore, further studies employing other network measures and parcellation schemes will provide more comprehensive insights into the ILA connectome.

## Conclusion

In summary, the brain connectomic alterations identified in this study provide further evidence to support the concept that ILA pathologies could disrupt brain connections, impairing network functioning, and cognition *via* a “disconnection syndrome.”

## Data Availability Statement

All datasets presented in this study are included in the article/Supplementary Material.

## Ethics Statement

The studies involving human participants were reviewed and approved by the Research Ethics Committee of Affiliated ZhongDa Hospital and the Southeast University. The patients/participants provided their written informed consent to participate in this study.

## Author Contributions

ZW and ZZ conceived and designed the research. YZ performed the experiments. QW, YW, XC, and YS collected the data. TL and CX provided technical assistance. ZW and YZ wrote the manuscript.

## Conflict of Interest

The authors declare that the research was conducted in the absence of any commercial or financial relationships that could be construed as a potential conflict of interest.

## Footnotes

^1^http://www.fil.ion.ucl.ac.uk/spm

^2^http://www.restfmri.net/forum/dparsf

^3^http://www.nitrc.org/projects/gretna/

^4^http://www.nitrc.org/projects/panda/

## Supplementary Material

FIGURE S1 Validation results with the “scrubbing” procedure for head motion correction in the preprocessing steps. To determine the extent to which our findings were robust to the motion correction strategy, we repeated the network centrality analyses using the scrubbed R-fMRI data and found that our main results identified in the primary analyses were not affected. Black asterisks (*) indicate a significant difference between the ILA and HC groups (permutation testing, *P* < 0.05). HC, healthy control; ILA, ischemic leukoaraiosis; R-fMRI, resting-state functional MRI.

FIGURE S2 Reproducibility of our principal findings using other parcellation schemes. We reconstructed the functional brain networks using the Dosenbach-160 atlases and found that our main results could be reproduced. Black asterisks (*) indicate a significant difference between the ILA and HC groups (permutation testing, *P* < 0.05). HC, healthy control; ILA, ischemic leukoaraiosis.
