# Supplementary figures and images for: Functional Disorganization of Small-World Brain Networks in Patients With Ischemic Leukoaraiosis
Source: Front Aging Neurosci. 2020 Jul 3;12:203. doi: 10.3389/fnagi.2020.00203 (PMC7348592; doi:10.3389/fnagi.2020.00203)

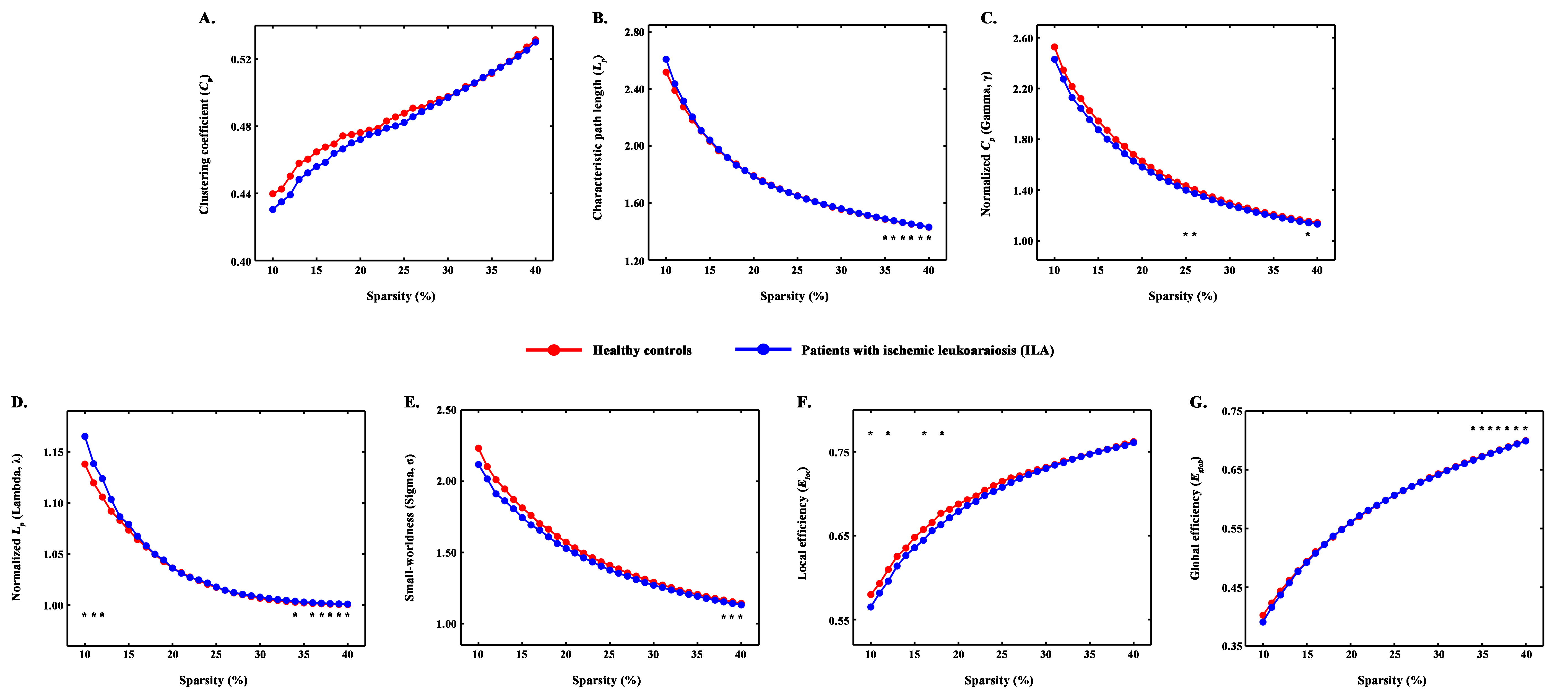

Supplement: FIGURE S1 — Validation results with the “scrubbing” procedure for head motion correction in the preprocessing steps. To determine the extent to which our findings were robust to the motion correction strategy, we repeated the network centrality analyses using the scrubbed R-fMRI data and found that our main results identified in the primary analyses were not affected. Black asterisks (*) indicate a significant difference between the ILA and HC groups (permutation testing, P < 0.05). HC, healthy control; ILA, ischemic leukoaraiosis; R-fMRI, resting-state functional MRI. [file Image_1.tif]

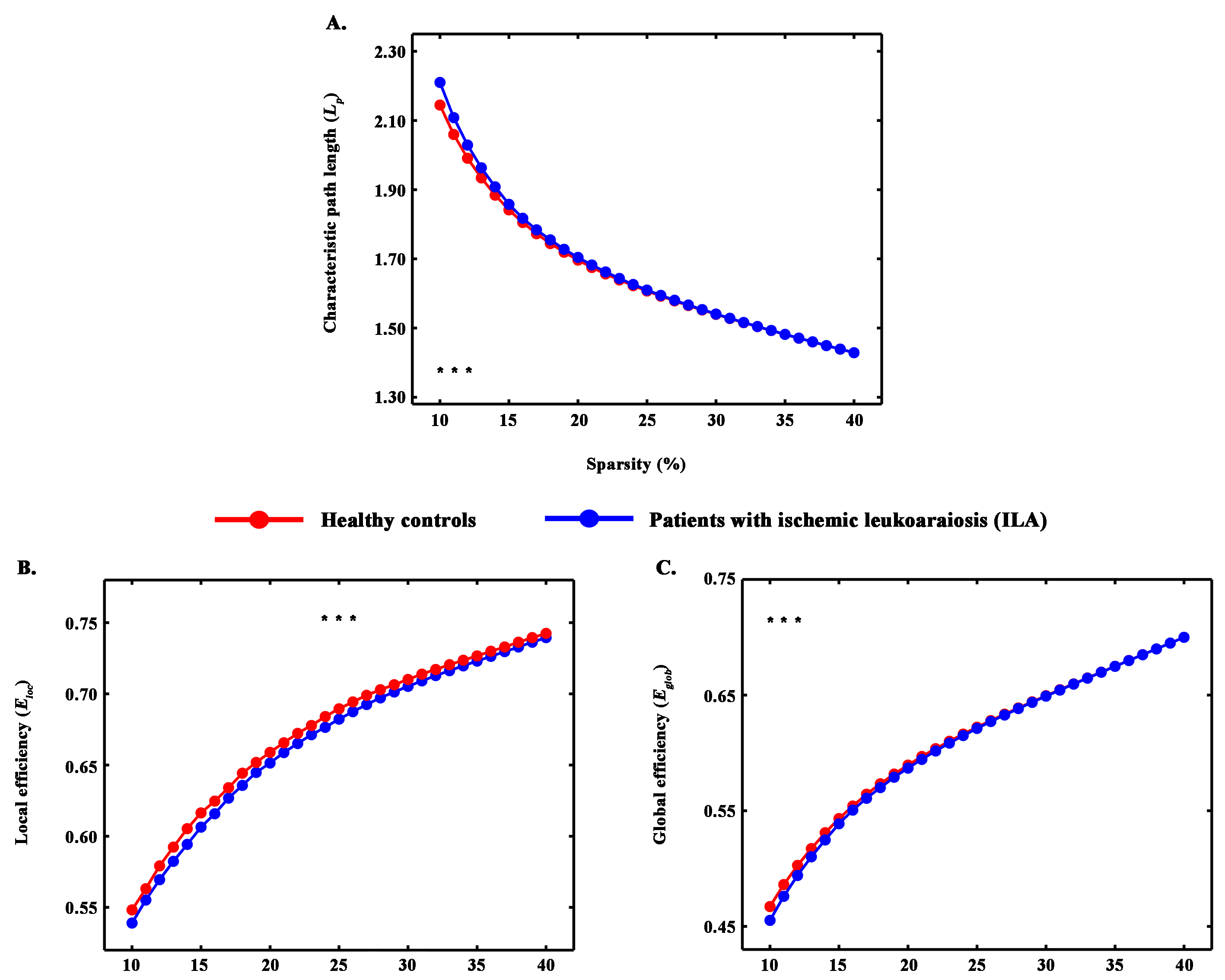

Supplement: FIGURE S2 — Reproducibility of our principal findings using other parcellation schemes. We reconstructed the functional brain networks using the Dosenbach-160 atlases and found that our main results could be reproduced. Black asterisks (*) indicate a significant difference between the ILA and HC groups (permutation testing, P < 0.05). HC, healthy control; ILA, ischemic leukoaraiosis. [file Image_2.tif]
